# Discovering the Repeatome of Five Species Belonging to the Asteraceae Family: A Computational Study

**DOI:** 10.3390/plants12061405

**Published:** 2023-03-22

**Authors:** Maria Ventimiglia, Marco Castellacci, Gabriele Usai, Alberto Vangelisti, Samuel Simoni, Lucia Natali, Andrea Cavallini, Flavia Mascagni, Tommaso Giordani

**Affiliations:** Department of Agriculture, Food and Environment (DAFE), University of Pisa, Via del Borghetto, 80-56124 Pisa, Italy

**Keywords:** Asteraceae, DNA transposons, repetitive DNA, retrotransposons, retrotransposon insertion time profile

## Abstract

Genome divergence by repeat proliferation and/or loss is a process that plays a crucial role in species evolution. Nevertheless, knowledge of the variability related to repeat proliferation among species of the same family is still limited. Considering the importance of the Asteraceae family, here we present a first contribution towards the metarepeatome of five Asteraceae species. A comprehensive picture of the repetitive components of all genomes was obtained by genome skimming with Illumina sequence reads and by analyzing a pool of full-length long terminal repeat retrotransposons (LTR-REs). Genome skimming allowed us to estimate the abundance and variability of repetitive components. The structure of the metagenome of the selected species was composed of 67% repetitive sequences, of which LTR-REs represented the bulk of annotated clusters. The species essentially shared ribosomal DNA sequences, whereas the other classes of repetitive DNA were highly variable among species. The pool of full-length LTR-REs was retrieved from all the species and their age of insertion was established, showing several lineage-specific proliferation peaks over the last 15-million years. Overall, a large variability of repeat abundance at superfamily, lineage, and sublineage levels was observed, indicating that repeats within individual genomes followed different evolutionary and temporal dynamics, and that different events of amplification or loss of these sequences may have occurred after species differentiation.

## 1. Introduction

The collection of all repetitive sequences distributed along chromosomes, known as the “repeatome”, constitutes one of the major components of eukaryotic genomes [1]. Overall, repeat types can be characterized as satellite DNA (i.e., sequences organized as tandem repetitions) and interspersed repeats (i.e., transposable elements) [2]. Transposable elements (TEs) are DNA sequences that can move independently within the genome through specific transposition mechanisms. The discovery of TEs dates back to the 1940s, when U.S. biologist Barbara McClintock identified DNA sequences capable of moving from one locus to another within the *Zea mays* genome [3]. Based on their transposition mechanism, TEs are divided into two main classes: retrotransposons (REs), or Class I TEs; and DNA transposons, or Class II TEs. Both classes are autonomous and non-autonomous elements based on the presence or absence of specific open reading frames encoding transposon proteins. Non-autonomous elements are not able to transpose autonomously but can still proliferate by exploiting the transposition proteins encoded by the autonomous elements [4,5,6,7]. DNA transposons can move through a mechanism of transposition called “cut-and-paste”, whereas retrotransposons use a “copy-and-paste” type of replication involving an intermediate RNA molecule [8]. REs can also be divided into two major groups based on the presence or absence of two directly oriented repeated sequences, called long terminal repeats (LTRs), which flank the element and are identical in newly transposed elements. Between the two LTRs is the coding region of the RE, which is organized into two sub-regions: *gag* and *pol*. The former contains a single gene encoding the capsid protein, which protects the system during the retrotranscription phase, while the latter encodes a polyprotein comprising the protein domains necessary for the replication and integration of the element into the host genome [9]. These domains include the following: a protease (PR) to cleave the polyprotein; a reverse transcriptase (RT), which synthesises the double strand from the single-stranded intermediate RNA template; an RNAseH (RH) to degrade the single-stranded RNA; and an integrase (INT), which is required for integration of the new element at the chosen genomic locus. The sequence order of the coding region defines the major superfamilies into which the LTR-REs are divided. In plants, LTR-REs can belong to two major superfamilies, *Gypsy* and *Copia*, which differ from each other in the position of a protein domain (INT) within the coding region [7]. In turn, the *Copia* and *Gypsy* superfamilies are subdivided into lineages that are distinguished based on the sequence similarity of the coding regions [10]. In Angiosperms, the most significant *Gypsy* lineages are the *Chromoviruses* (in particular, *Galadriel*, *Tekay*, *Reina*, *CRM*), characterized by the presence of the chromodomain at the 3′ end of the coding sequence, and the *non-Chromoviruses* (*Athila*, *Tat*, *Ogre* and *Retand*), which do not present the chromodomain. The main *Copia* lineages are *Ale*, *Ivana*, *Ikeros*, *Tork*, *Alesia*, *Angela*, *Bianca*, *SIRE*, and *TAR* [10]. Full-length LTR-REs range in size from a few hundred bases to 10 kb, including both autonomous and non-autonomous elements, and constitute the most abundant and variable group of TEs in plant genomes. In fact, in some plants, LTR-REs represent a major portion of the nuclear genome, with percentages of more than 50% [11].

TEs have long been referred to as “selfish” or “parasitic” DNA [12] because of their ability to “colonize” the genome, increasing their copy number using the metabolic tools of the host. In contrast, higher organisms have evolved systems of regulation and control (e.g., DNA methylation) that aim to limit TE expansion [13]. The role of TEs has been significantly re-evaluated, as it is speculated that they may have contributed to genome remodelling through mechanisms such as gene duplication, exon shuffling, and novel gene formation, actively contributing to genetic diversity and adaptation [14,15]. Today, TEs are often defined as symbiotic partners of the host, whose activity can have neutral, favourable, or harmful consequences for the host genome [16,17].

Most variations in genome structure and evolution reflect the dynamics of the proliferation and loss of TEs [18]. In plants, these phenomena have been studied mostly on small- or medium-sized genomes and on a few large-sized genomes, such as monocotyledonous species maize [19] and barley [20]. For dicotyledonous plants, in *Helianthus*, a widely studied genus characterized by large genomes shows significant variability among repetitive components [21].

The genome of *Helianthus annuus* is composed of over 81% TEs, and REs (especially LTR-REs) are the most abundant class of sequences, accounting for at least 77% of them [22,23,24]. Despite their economic importance, the genome composition and organisation of other Asteraceae species are largely unknown. However, Asteraceae genomes differ in the abundance and diversity of TEs [25].

Considering other important crops, such as lettuce and artichoke, together with officinal and ornamental species (i.e., *Artemisia annua* and *Chrysanthemum seticuspe*), we exploited different genomic resources to construct a “metarepeatome” belonging to five different species, providing new possibilities for studying the structure of genomes and allowing the investigation of many aspects, including the dynamics and changes in the repetitive genomic components among Asteraceae.

The identification of repetitive elements using graph-based clustering of short sequence reads [26] is one of the most frequently used bioinformatics tools in genome skimming [27], specifically designed to exploit the potential of NGS technologies, and it has appeared efficient in characterizing the repetitive components of plants [21,24,28,29]. This de novo approach could be particularly useful in discovering repeats that are difficult to identify with structural tools. The identification of repeats based only on structural features, in fact, could lead to mismeasurements of repeat abundance. Repeat sequences in species where transposition events occurred in very ancient times could have accumulated mutations and have been poorly detected. Furthermore, scanning genome sequences for identifying full-length elements could result in a low number of repetitive elements because of common mis-assembly events (i.e., repeats collapsing during the assembly procedure) [30].

Based on graph clustering and the identification of full-length repeats, this study aimed to clarify the repeatome belonging to important Asteraceae and shed light on various evolutionary and temporal dynamics of retrotranspositional activity following species separation by: (i) Establishing the extent of repetitive DNA variation among species belonging to the same family; and (ii) Analyzing the relationship between changes in LTR-RE abundance and variations in the dynamics of specific LTR-REs among related species.

## 2. Results

### 2.1. Metarepeatome Analysis of Asteraceae Species

The repeatomes of five species of the Asteraceae family (i.e., *Helianthus annuus*, *Lactuca sativa*, *Cynara cardunculus* var. *scolymus*, *Artemisia annua*, and *Chrysanthemum seticuspe*) were studied to classify repetitive sequences and identify their homologous groups in individual genomes (Table 1).

A comparative analysis using hybrid clustering was performed with RepeatExplorer2 using a set of 1,000,000 random reads from each of the five chosen species for a total of 5,000,000 reads. The clustered sequence reads, i.e., the repetitive DNA, ranged from 60.44% of the genome of *Cynara cardunculus* var. *scolymus* to 78.44% of the genome of *Helianthus annuus* (Table 2).

**Figure 1:**
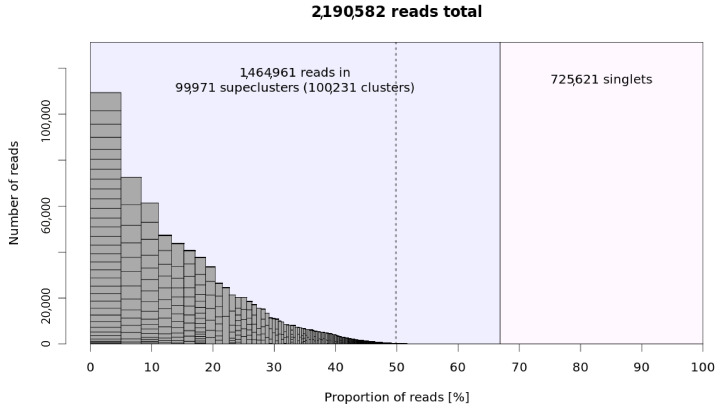
Graphical summary of hybrid clustering results. The bars represent superclusters, with their heights and widths corresponding to the number of reads in the superclusters (y-axis) and their proportions in all analyzed reads (x-axis), respectively. The rectangles within the supercluster bars represent the individual clusters. The blue and pink background panels show the proportions of reads that have been clustered and those that have remained single, respectively. The top clusters are to the left of the dotted line.

In total, 2,190,582 reads were grouped into 100,231 clusters, representing different subfamilies of specific repetitive elements. Furthermore, exploiting the feature of paired-end reads, clusters were grouped into 99,971 superclusters, which included repeats belonging to the same repeat family. In total, this analysis estimated the repetitive component as 67% of the metagenome structure of the five species, while 725,621 sequences remained singlets (Figure 1).

**Figure 2:**
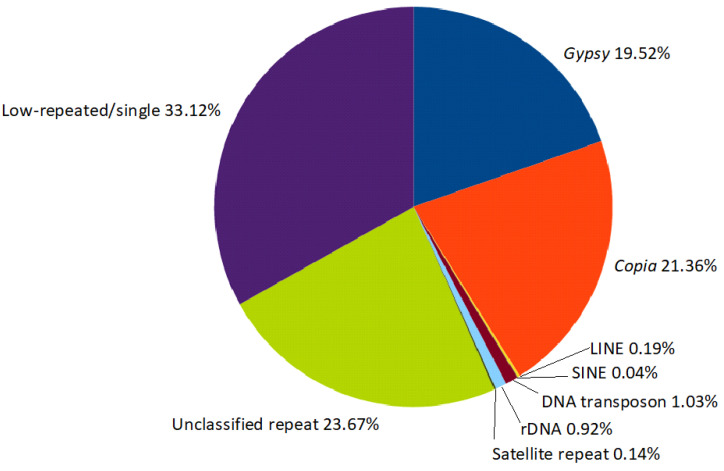
The composition of the metarepeatome of the five Asteraceae species evaluated.

Of the 528 top clusters (i.e., clusters representing >0.01% of the analyzed reads), 455 were annotated as repeats belonging to the LTR order, showing that the overall structure of the five species was largely composed of LTR-RE-related clusters. Among the clusters annotated as LTR-REs, the two major superfamilies were represented by similar percentages: 21.36% and 19.52% of the metarepeatome for *Copia* and *Gypsy*, respectively. DNA transposons accounted for 1.03% of the metarepeatome, rDNA sequences for 0.92%, and satellite DNA for 0.14%. Finally, 23.67% consisted of unidentifiable repeated elements, and 33.12% was attributable to single or low-copy-number sequences, including repeats that were not abundant in the respective species (Figure 2).

Concerning LTR-retrotransposons (Table 3), the repeats annotated as LTR-REs ranged from 35.27% of the genome of *Cynara cardunculus* var. *scolymus* to 52.32% in *Chrysanthemum seticuspe*. *Gypsy* elements ranged from 10.61% in *Cynara cardunculus* var. *scolymus* to 41.11% in *Helianthus annuus*, whereas *Copia* elements ranged from 6.35% in *Helianthus annuus* to 35.28% in *Chrysanthemum seticuspe*. The ratio between the genomic proportions of *Gypsy* and *Copia* elements largely differed among these Asteraceae species, from 0.35 in *Chrysanthemum seticuspe* to 6.47 in *Helianthus annuus* (Table 3). The maximum difference of genome proportion of each LTR-RE superfamily or lineage among the five species analyzed gave us an estimation of genome proportion variability of *Copia* and *Gypsy* elements within Asteraceae. Such variability among genomes was larger for each *Gypsy* lineage compared to *Copia* lineages, and it was even larger for whole superfamilies; the maximum difference was 28.93% for the *Copia* superfamily and 30.50% for the *Gypsy* superfamily (Table 3). LTR-RE redundancy was also studied after annotating elements at the lineage level: six lineages (plus one group that could not be annotated) were identified among *Copia* REs (*Ale*, *Angela*, *Ikeros*, *Ivana*, *SIRE*, and *TAR*), and three lineages (plus one group that could not be annotated) were identified among *Gypsy* REs (*Chromovirus*, *Athila* and *Tat*) (Table 3). Among the *Copia* REs, the *SIRE* lineage had a genome proportion higher than 3% in all species, while the *Angela* lineage was particularly abundant (14.66%) in *Lactuca sativa*. Each *Gypsy* lineage accounted for different percentages of the genome, with *Chromoviruses* being the most abundant, especially in *Helianthus annuus* (31.11%).

**Figure 3:**
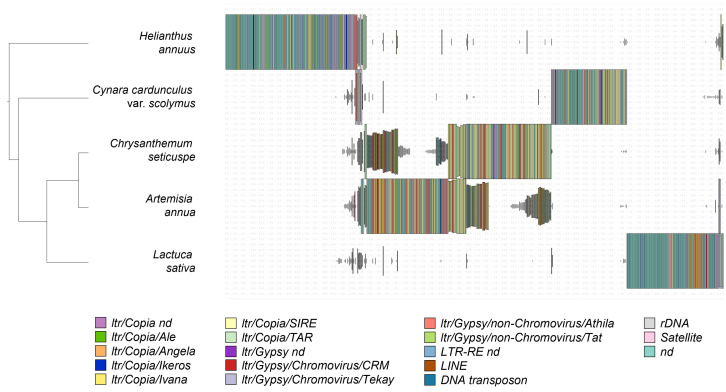
Comparison of hybrid clustering results among the five Asteraceae species. The bars represent the genome proportion of each cluster for each species; a legend is reported to indicate the repeat class, superfamily, or lineage. On the left, groups of clusters are labelled as assessed by hierarchical clustering of the results.

To investigate the possible variability within lineages and to identify species-specific repeats, hierarchical clustering was performed on the annotated clusters based on their abundance within the analyzed genomes and grouping the homologous shared clusters. As shown in Figure 3, the analyzed species essentially shared rDNA sequences. The other DNA repeat classes were very specific, with the presence of distinct sublineages, except for *Artemisia annua* and *Chrysanthemum seticuspe* (which belong to the same tribe, Anthemideae, and share some of their repeats).

### 2.2. Isolation and Analysis of Full-Length LTR Retrotransposons

Because LTR-REs are largely the most abundant repeat class in the genomes of the five Asteraceae species, full-length LTR-REs were identified based on the structural features in the sequenced genomes of each selected species. Overall, 48,872 full-length LTR-REs were retrieved (Table 4).

Most of the full-length LTR-REs (34,580 out of 48,872) were identified in the large genome of *Helianthus annuus*, 77.5% of which were annotated as *Gypsy*-related LTR-REs, with a prevalence of elements belonging to the *Chromovirus*/*Tekay* lineage. Then, 6875 full-length LTR-REs were found in lettuce, 79% of which belonged to the *Copia* superfamily.

**Figure 4:**
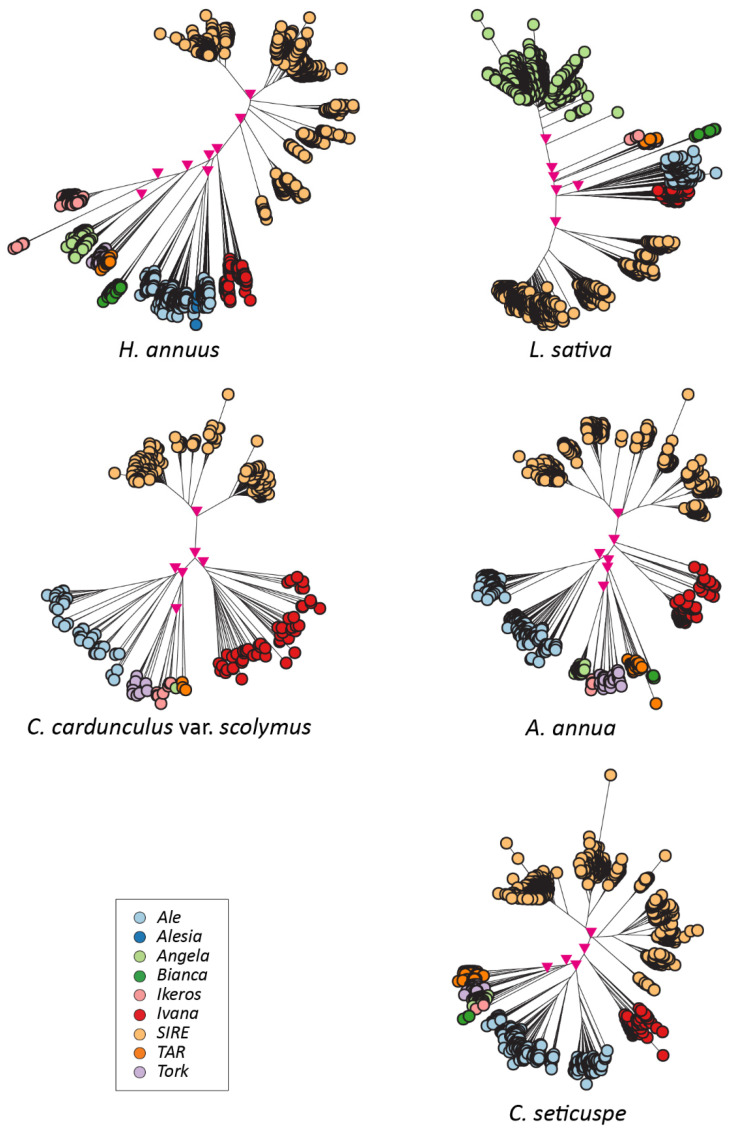
Phylogenetic trees of the LTR-REs of the *Copia* superfamily in individual species. The main nodes (bootstrap values > 0.6) separating the lineages are marked with pink triangles.

**Figure 5:**
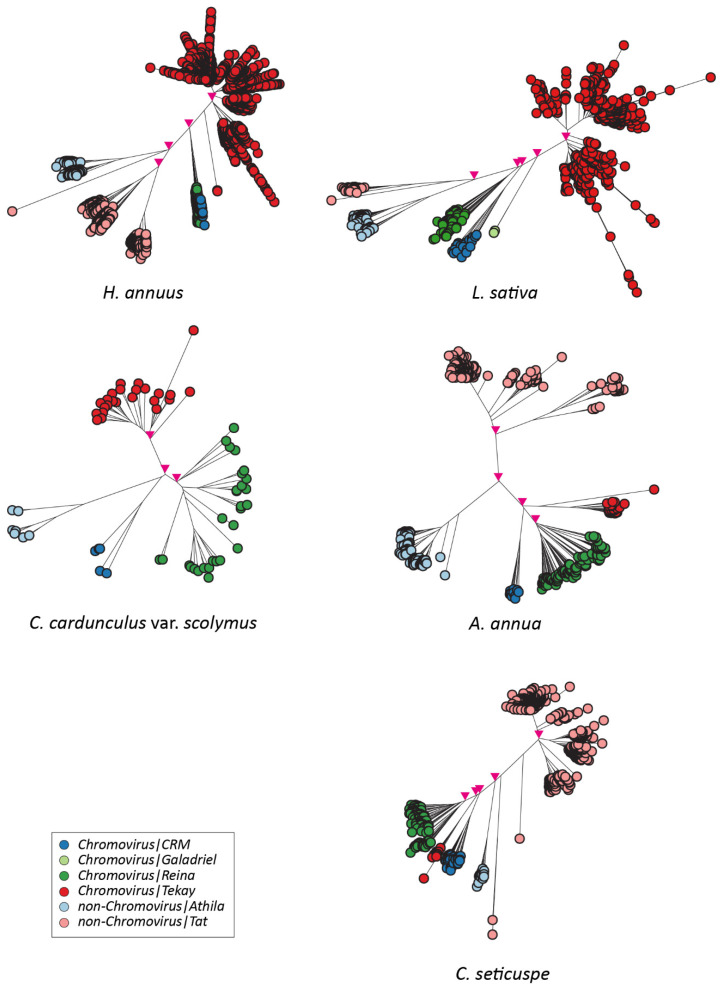
Phylogenetic trees of the LTR-REs of the *Gypsy* superfamily in individual species. The main nodes (bootstrap values > 0.6) separating the lineages are marked with pink triangles.

The sequences encoding RT domains (15,431 intact RT domains for the *Copia* superfamily and 26,203 intact RT domains for the *Gypsy* superfamily) were identified and collected from the pool of full-length elements and analyzed to infer the phylogenetic relationship occurring among the LTR-REs of a single species, highlighting a clear separation of lineages in each of the studied genomes (Figure 4 and Figure 5).

Furthermore, the phylogenetic trees based on all RT sequences of *Copia* and *Gypsy* elements, separated according to the lineage, revealed that for all lineages, RT sequences clustered randomly, i.e., not based on the species to which they belonged (Supplementary Figures S1 and S2).

**Figure 6:**
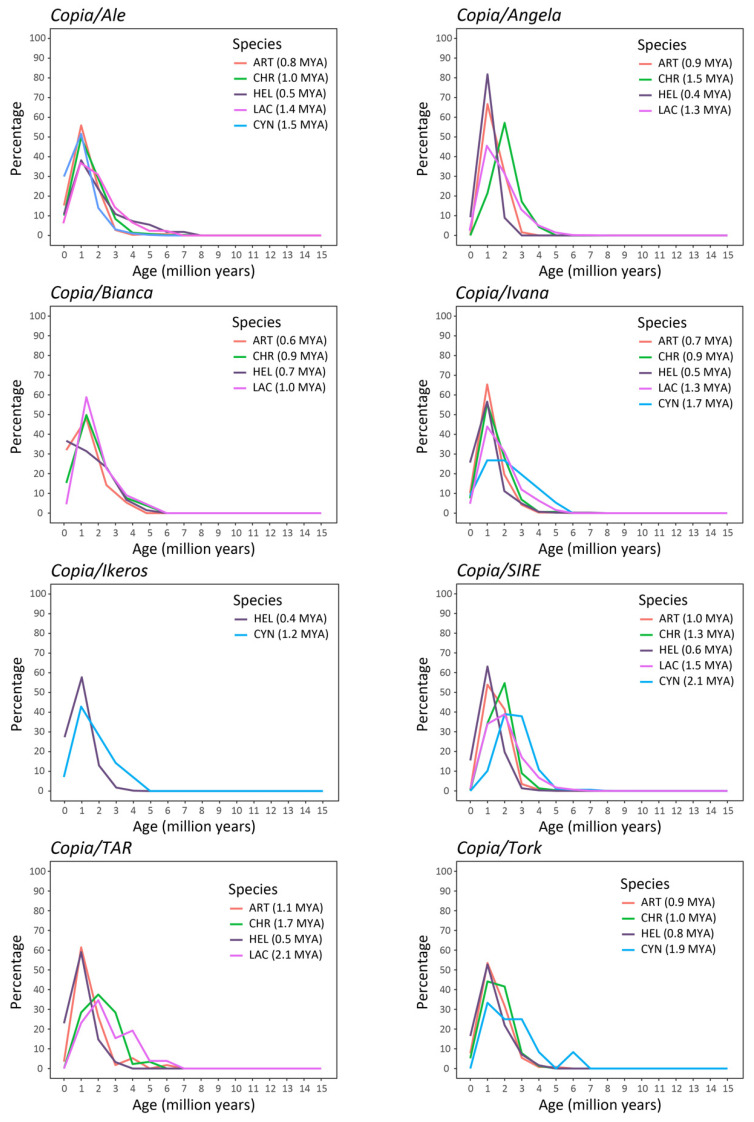
Insertion time of *Copia* elements in the five Asteraceae species. The average insertion time (in MYA) for each species is reported in parentheses. HEL = *Helianthus annuus*, LAC = *Lactuca sativa*, CYN = *Cynara cardunculus* var. *scolymus*, ART = *Artemisia annua*, CHR = *Chrysanthemum seticuspe*.

**Figure 7:**
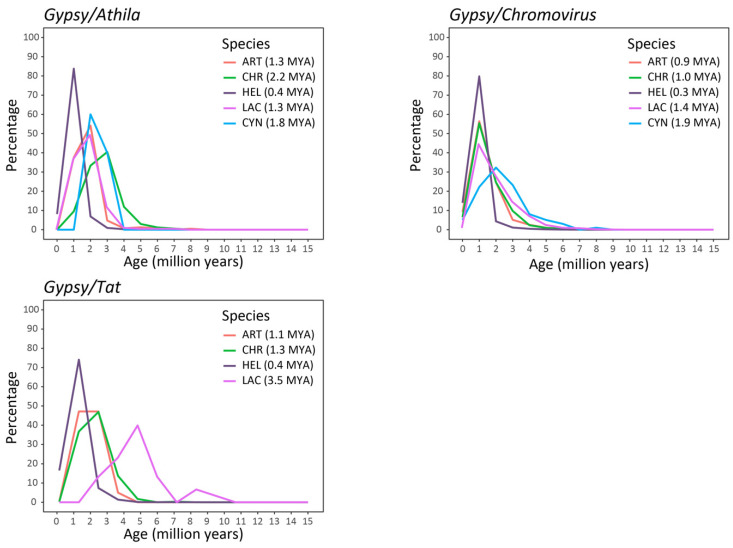
Insertion time of *Gypsy* elements in the five Asteraceae species. The average insertion time (in MYA) for each species is reported in parentheses. HEL = *Helianthus annuus*, LAC = *Lactuca sativa*, CYN = *Cynara cardunculus* var. *scolymus*, ART = *Artemisia annua*, CHR = *Chrysanthemum seticuspe*.

Finally, proliferation time profiles of the full-length LTR-REs were analyzed in the five genomes by measuring pairwise distances between the LTRs of the same element. The two LTR sequences of a retrotransposon are identical immediately after the insertion event and then undergo mutations over time [36]. If LTR-REs accumulate more mutations than genes as time passes [21], distances between LTR sequences are converted into timing profiles using a mutation rate that is twice the rate calculated for synonymous substitutions in *Helianthus annuus* gene sequences [37,38]. This analysis showed the proliferation of LTR-REs in the last 15 MY (Figure 6 and Figure 7). The species presented different insertion time profiles specific to the different lineages. Most of the lineages of the *Copia* superfamily showed a proliferation peak at about 1 MYA (Figure 6), except for elements belonging to some lineages (*SIRE*, *TAR* and *Tork*) that showed older proliferation peaks in certain species, such as *Lactuca sativa* and *Cynara cardunculus* var. *scolymus*. The lineages belonging to the *Gypsy* superfamily were generally older and showed abundant proliferation activity between 1 and 5 MYA (Figure 7). Appreciable differences were also found by studying the proliferation events of the different lineages in the individual species. In *Helianthus annuus,* all but one lineage of the *Copia* superfamily revealed proliferation peaks around 1 MYA, while the *Bianca* lineage still appeared to be going through proliferation events, showing an upward curve (Figure 6). In *Lactuca sativa*, the *Gypsy* lineages *Chromovirus* and *Athila* showed proliferation peaks around 1 and 2 MYA, respectively, while *Tat* elements seemed older, with two different peaks around 8 and 5 MYA (Figure 7).

## 3. Discussion

The Asteraceae family is of considerable economic importance, and *Helianthus* has been a model system for studying the genetic mechanisms of speciation, hybridization, and domestication for more than two decades [39]. However, the characterization and possible involvement of the repeatome of other Asteraceae genomes in evolutionary processes are still poorly studied.

Repetitive sequences have been identified and quantified by hybrid graph-based clustering [26], a strategy commonly used to gain insight into the composition and sequence variation of repetitive components in a pool of related species [21,40,41]. Among the five selected Asteraceae species, repetitive DNA ranged from 60.44% in *Cynara cardunculus* var. *scolymus* to 78.44% in *Helianthus annuus*, similar to what has already been reported for this species by Giordani [42]. On the other hand, differences in transposable elements abundance were observed in the selected species comparing to previous studies [22,32,33,35,43]. Such variability can be due to the different genotypes analyzed, as reported in sunflower [24], or to the usage of diverse methods of repeat discovery and quantification. Clustering analyses, using unassembled reads obtained from low-coverage genome sequencing for estimating the genome proportion of the repeated sequences, is one of the most reliable methods as it has been demonstrated in other study systems [44,45,46].

The genome structure was similar among the analyzed species, with LTR-REs representing the most repetitive sequences. The prevalence of LTR-retrotransposons in the fraction of highly repeated sequences is a common feature of higher plant genomes, where retroelements represent one of the major forces driving genome size evolution [47,48,49] and were previously observed in Asteraceae by Staton [50]. However, striking differences in abundance and variability were observed after analyzing the different LTR-REs from the superfamily to the lineage level.

The ratio between the abundance of *Gypsy*- and *Copia*-related sequences was highly variable, ranging from 0.35 in the chrysanthemum to 6.47 in the sunflower. The TE abundance biased towards *Gypsy* TEs was observed in Asteraceae by Staton [50], suggesting that the two superfamilies have contributed differently to the genome community. Generally, in Angiosperms, *Gypsy* elements are more abundant than *Copia* elements, with valuable exceptions, such as pear, date palm, and banana [11]. However, this ratio is not apparently related to the taxonomy of species. The large variability of this ratio among the selected Asteraceae species confirms the data reported for higher plants (Angiosperms and Gymnosperms [11]) at the intrafamily level.

At the lineage level, among *Copia* lineages, *SIRE* elements were by far the most abundant in all analysed species, varying from 3.88% in lettuce to 29.91% (i.e., more than 7-fold) in chrysanthemum. Regarding *Gypsy* lineages, *Chromovirus* elements were the most frequent in the genomes, and their abundance varied from 2.07% in chrysanthemum to 30.42% (i.e., more than 14-fold) in sunflower. The predominance of *SIRE* and *Chromovirus* elements has also been observed in other Asteraceae genera, including *Hieracium* [45], *Senecio* [46], and *Stevia* [51] These variations indicate that the high amplification rate was maintained in certain species even after speciation or that other rearrangements, such as duplications of chromosomal fragments, may have occurred, producing such large variations. These results suggest that after species separation, the repetitive components underwent different rates of amplification/loss but also that new LTR-RE sublineages originated (by mutations or by horizontal transfer) in the genomes. This is because DNA repeats can co-evolve but also have a different and independent evolution with respect to the genome of the host [4].

The hybrid clustering of Illumina short reads from five species also provided information about an “average” composition of the analysed genomes, showing the extent of sharing repetitive sequences within this family.

On average, repetitive DNA represented about 67% of this “metagenome”. However, most of this repetitive fraction was comprised of repeats specific to each species, i.e., most repeats were not shared between Asteraceae species. In this sense, only the most abundant repeats of each species were represented in the clusters of the metagenome.

Moreover, the analyzed species shared ribosomal DNA sequences, while the other classes of repetitive DNA were generally species-specific. The exceptions were *Artemisia annua* and *Chrysanthemum seticuspe*, both belonging to the tribe Anthemideae, which shared several repeat clusters.

The dendrogram obtained by hierarchical clustering analysis (Figure 3) did not recapitulate the phylogenetic relationship between the five species, except for the two species belonging to the same tribe (*A. annua* and *C. seticuspe*), for which the dendrogram was consistent with the Asteraceae phylogeny. This suggests that the evolution of LTR-REs was partially independent of the evolution of such species, and that individual genomes have undertaken different evolutionary dynamics in the composition and abundance of repeated elements following speciation. This aspect is not surprising given the potential autonomy of these elements in replication within the host genomes [4].

Other analyses were performed to identify and characterize full-length elements belonging to the LTR-RE fraction of the repetitive DNA, i.e., the most abundant REs in the genome of each selected species, using the available genome assemblies (at both chromosome and scaffold levels).

Overall, 48,872 full-length LTR-REs were retrieved from the five analyzed species. Most of the full-length LTR-REs, about 71%, were isolated in sunflower, the species with the largest genome (3.6 Gbp) [31] and the largest abundance in repeats [24]. However, many full-length elements were identified and characterized for the first time in the other Asteraceae species evaluated in this study.

The isolation of full-length LTR-REs enabled us to obtain important information about the variability and phylogeny of REs within the studied genomes. Indeed, full-length LTR-REs present highly conserved domains that may preserve their functionality and allow effective reconstruction of the evolutionary dynamics that lead to the differentiation of the repeatomes within Asteraceae.

The phylogenetic trees showed a well-defined clustering of RT-encoding sequences according to the LTR-RE lineages within each species (Figure 4 and Figure 5), indicating that LTR-RE lineage separation occurred before Asteraceae speciation.

However, in RT-related dendrograms constructed by separating LTR-RE lineages (Supplementary Figures S1 and S2), the separation among species was less defined, suggesting that different sublineages had undergone different transposition rates after speciation.

Finally, a large variability was also observed concerning the temporal profiles of transposition bursts, established by comparing LTR sequences of isolated full-length elements [36]. As a result of the amplification burst(s) that may have occurred, our data on the LTR-RE insertion age (Figure 6 and Figure 7) demonstrate that RE amplification occurred at different times for different species.

## 4. Conclusions

Our study exploits the potentiality of massive parallel sequencing technologies applied to the analysis of genome structure and evolution, representing a first contribution towards the metarepeatome of the Asteraceae family. The identification and characterization of repeat sequences in these species will aid in genome annotation, as well as in the development of molecular markers for breeding programs. Overall, a large variability of repeat abundance at superfamily, lineage, and sublineage levels was observed, suggesting that the repeatomes within individual genomes followed different evolutionary and temporal dynamics, indicating that different events of amplification or a loss of most LTR-RE lineages occurred after species separation. This is in line with studies highlighting the potential autonomous nature of repeats [4]: cases of species-specific huge amplification of LTR-RE lineages were already reported in sunflowers [52,53], where LTR-REs were identified as retrotranspositionally active [54]. Further analyses related to the mobility of retrotransposons will be useful to define with more precision the evolution of the repetitive component along the selected genomes, knowing that LTR-REs can affect not only the coding portion of the genome but also modify the cis-regulatory sequences of the genes, with possible heritable phenotype changes in plant species.

## 5. Materials and Methods

### 5.1. Sequence Data Collection

After exploring the data available in the NCBI GenBank, five economically relevant species of the Asteraceae family were chosen. In particular, the genome assembly and read packages produced by NGS Illumina sequencing techniques of *Helianthus annuus*, *Lactuca sativa*, *Cynara cardunculus* var. *scolymus*, *Artemisia annua*, and *Chrysanthemum seticuspe* were selected and downloaded.

FastQC v0.11.5 [55], software embedded in the Galaxy platform of RepeatExplorer2 [56], was used to perform sequence quality checks of the FASTQ-formatted read packages. At the end of the process, the software provided a quality report. Trimming by Trimmomatic v0.39 [57] was performed based on the quality control results to clean up the read datasets and to make subsequent analyses easier and more accurate. Using this tool, reads with a low-quality score were discarded, and adapters were removed. All reads containing organellar DNA sequences were removed using CLC–BIO Genomic Workbench 9.5.3 (CLC-BIO, Aarhus, Denmark) against a library consisting of the chloroplast sequences of the five Asteraceae species (NCBI codes: MK341452.1, *Helianthus annuus*; AP007232.1, *Lactuca sativa*; KP842713.1, *Cynara cardunculus* var. *scolymus*; PKPP01000155.1, *Artemisia annua*; NC_040920.1, *Chrysanthemum lucidum*) and the mitochondrial sequence of *Helianthus annuus* (NCBI code: CM007908).

### 5.2. Clustering Analyses with RepeatExplorer2

The reads of all five Asteraceae species, processed as above, were used to perform hybrid clustering with RepeatExplorer2. A total of 1,000,000 reads (forward and reverse) extracted from the input files of each species were used for this analysis. The resulting clusters were built by an all-to-all comparison of sequence reads to reveal their similarities and represent different repetitive element subfamilies. This tool also provided a list of superclusters, i.e., clusters of shared paired-end reads representing the same repeat family.

Similarity searches by blastn and tblastx, using the BLAST package v2.6.0+ [58] with default parameters, were performed on the remaining unknown clusters against a library of repetitive sequences belonging to sunflower, SUNREP [23], to increase the number of annotated clusters.

### 5.3. Identification and Characterisation of Full-Length LTR-REs

Full-length LTR-REs were identified in the five Asteraceae genomes using LTRharvest (GenomeTools v1.5.10, options: -minlenltr 100—maxlenltr 10,000 -mindistltr 1500 -maxdistltr 25,000 -mintsd 5 -maxtsd 5 -motif tgca -vic 10) [59]. The identified sequences were initially annotated using LTRdigest (GenomeTools v1.5.10) [60] and then submitted to the DANTE tool v1.1.0 provided on the RepeatExplorer Galaxy-based website (https://repeatexplorer-elixir.cerit-sc.cz/galaxy/, accessed on 27 October 2022). The annotations obtained were thus checked through an in-house-built Python script to identify and remove nested elements (i.e., when a TE insertion occurs into an existing TE) and those elements showing an inappropriate number and/or order of protein domains to create a final annotation. The LTR-REs were classified at the superfamily and lineage levels, according to Neumann [10].

### 5.4. Phylogenetic Analysis of LTR-REs

The pool of LTR-REs was analysed to isolate sequences corresponding to the reverse transcriptase (RT) protein domains. The RT domain was chosen because it represents a protein region essential for the transposition process (present in both superfamilies) and is, therefore, conserved among species. The sequences were aligned using MAFFT v7.475 [61], and then ClustalW v2.1 [62] was used to build neighbour-joining (NJ) trees. The NJ trees were edited with R software [63]. The robustness of the trees was tested by repeated random resamplings for 100 interactions. Phylogenetic trees were constructed by separating the species or LTR-RE lineages.

### 5.5. Evaluation of the Insertion Time of LTR-REs

The age of insertion of the LTR-REs was estimated by comparing the LTR sequence at the 5′ end and the LTR sequence at the 3′ end of each full-length element [36]. The two LTRs of each element were first aligned using the Stretcher tool (EMBOSS package v6.6.0.0) [64], and then the nucleotide distances between the LTRs were measured using the Kimura two-parameter method (K2P) [65] implemented in the Distmat tool (EMBOSS package) [64] using an in-house built perl script. The K2P method is one of the most widely used mathematical models for predicting nucleotide substitutions, i.e., mutations caused by exchanging one nucleotide with another. For the analyzed sequences, the Kimura distances were converted to MYA using a synonymous substitution rate that is twice that calculated for sunflower genes, i.e., 2 × 10^−8^ [21].

## Figures and Tables

**Table 1:** Data on Asteraceae genome assemblies and Illumina read packages used.

Species	Common Name	GenBank Assembly Accession	Assembly Level	SRA ID	Raw Paired-End Reads	Trimmed Reads (100 bp)
*Helianthus annuus*	Sunflower	GCA_002127325.2 [31]	Chromosome	SRR5004633	124,824,626	82,204,512
*Lactuca sativa*	Lettuce	GCA_002870075.2 [32]	Chromosome	SRR577192	187,005,846	117,409,692
*Cynara cardunculus* var. *scolymus*	Globe artichocke	GCA_001531365.1 [33]	Chromosome	SRR1914381	91,528,290	73,595,420
*Artemisia annua*	Annual mugwort	GCA_003112345.1 [34]	Scaffold	SRR5602595	1,330,400	1,076,116
*Chrysanthemum seticuspe*	Chrysanthemum	GCA_004359105.1 [35]	Scaffold	DRR087118	382,227,342	330,102,622

**Table 2:** Total read count, number of clustered reads and corresponding genome proportion for each species, as obtained by the comparative analysis of hybrid clustering results.

Species	Total Read Count [Nr]	Reads in Cluster [Nr]	Genome Proportion [%]	Genome Size [Gb]
*Helianthus annuus*	438,456	343,922	78.44	3.6
*Lactuca sativa*	438,358	280,372	63.96	2.5
*Cynara cardunculus* var. *scolymus*	437,906	264,665	60.44	1.07
*Artemisia annua*	438,250	273,986	62.52	1.74
*Chrysanthemum seticuspe*	437,612	302,014	69.01	3.06

**Table 3:** Genome proportion of LTR-RE sequences, expressed as percentage, and maximum difference among the five Asteraceae species. LTR-RE = long terminal repeat retrotransposon.

LTR-RE	Superfamily	Lineage	*Helianthus annuus*	*Lactuca sativa*	*Cynara cardunculus* var. *scolymus*	*Artemisia annua*	*Chrysantemum seticuspe*	Maximum Difference
	***Copia***	*Ale*	0.00	0.00	0.00	0.00	0.17	0.17
		*Angela*	0.07	16.99	0.01	2.04	4.01	16.99
		*Ikeros*	0.29	0.00	0.00	0.00	0.00	0.29
		*Ivana*	0.00	0.00	0.00	0.23	0.10	0.23
		*SIRE*	5.57	3.88	23.49	16.04	29.91	26.03
		*TAR*	0.11	0.03	0.01	0.44	0.72	0.71
		Unknown	0.31	0.95	0.00	1.05	0.38	1.05
		Total *Copia*	6.35	21.86	23.50	19.80	35.28	28.93
	***Gypsy***	*Chromovirus*	30.42	12.9	9.66	3.62	2.07	28.35
		*Athila*	3.05	0.55	0.95	15.46	9.38	14.90
		*Tat*	5.25	0.01	0.00	0.90	0.80	5.25
		Unknown	2.39	0.20	0.00	0.00	0.00	2.39
		Total *Gypsy*	41.11	13.67	10.61	19.98	12.25	30.50
	Unknown		4.24	12.59	1.15	3.06	4.79	11.44
**TOTAL**			51.70	48.12	35.27	42.84	52.32	17.05
***Gypsy*/** ***Copia***			6.47	0.63	0.45	1.01	0.35	

**Table 4:** Number of LTR-REs identified for each genome, specified for each superfamily and lineage. LTR-RE = long terminal repeat retrotransposon.

Lineage	*Helianthus* *annuus*	*Lactuca* *sativa*	*Cynara cardunculus* var. *scolymus*	*Artemisia* *annua*	*Chrysanthemum seticuspe*
*Ale*	674	208	55	288	630
*Alesia*	9	15	0	0	0
*Angela*	312	2278	1	63	70
*Bianca*	133	22	0	56	26
*Ikeros*	505	5	14	8	2
*Ivana*	400	125	56	323	304
*SIRE*	4711	2493	176	630	1284
*TAR*	61	26	4	57	88
*Tork*	182	8	12	129	77
*Copia* unclassified	656	246	19	380	602
*Copia* total	7643	5426	337	1934	3083
*Chromovirus|CRM*	119	46	5	30	47
*Chrommovirus|Galadriel*	3	3	0	0	0
*Chromovirus|Reina*	235	65	44	123	146
*Chromovirus|Tekay*	18,405	1027	48	35	21
*Chromovirus* unclassified	424	13	-	5	1
*non-Chromovirus|OTA|Athila*	2472	213	10	398	168
*non-Chromovirus|OTA|Tat*	5060	30	0	260	697
*non-Chromovirus|OTA* unclassified	7	-	-	4	1
*non-Chromovirus* unclassified	-	-	-	-	-
*Gypsy* unclassified	84	-	-	1	-
*Gypsy* total	26,809	1397	107	856	1081
LTR-RE unclassified	128	52	4	9	6
TOTAL	34,580	6875	448	2799	4170

## Data Availability

Publicly available datasets were analyzed in this study. This data can be found here: https://www.ncbi.nlm.nih.gov/ (accessed on 27 October 2022).

## Supplementary Materials

The following supporting information can be downloaded at: https://www.mdpi.com/article/10.3390/plants12061405/s1, Figure S1: Phylogenetic trees of all RT sequences of *Copia* elements retrieved in the five Asteraceae species, separated according to the lineage; Figure S2: Phylogenetic trees of all RT sequences of *Gypsy* elements retrieved in the five Asteraceae species, separated according to the lineage.
