# Supplementary material for: Discovering the Repeatome of Five Species Belonging to the Asteraceae Family: A Computational Study
Source: Plants (Basel). 2023 Mar 22;12(6):1405. doi: 10.3390/plants12061405 (PMC10058865; doi:10.3390/plants12061405)
Supplement: Supplementary file 1 [file plants-12-01405-s001.zip › plants-2238961-supplementary.pdf]

plants

Supplementary Materials

Discovering the Repeatome of Five Species Belonging to the Asteraceae Family: A Computational Study

Maria Ventimiglia †, Marco Castellacci †, Gabriele Usai, Alberto Vangelisti, Samuel Simoni, Lucia Natali, Andrea Cavallini, Flavia Mascagni * and Tommaso Giordani

Department of Agriculture, Food and Environment (DAFE), University of Pisa, Via del Borghetto, 80-56124 Pisa, Italy
* Correspondence: flavia.mascagni@unipi.it
† These authors contributed equally to this work.

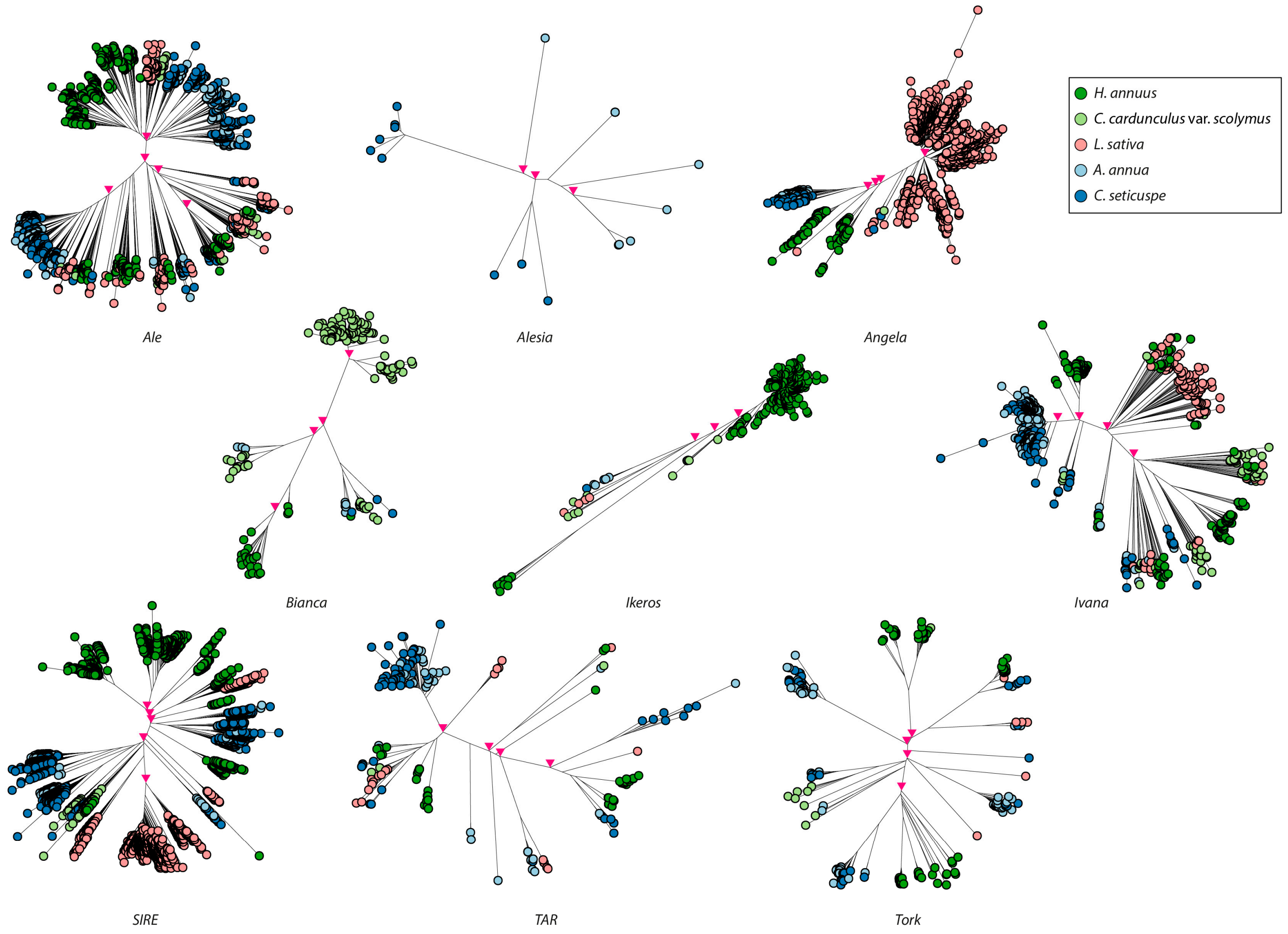

Figure S1. Phylogenetic trees of all RT sequences of *Copia* elements retrieved in the five Asteraceae species, separated according to the lineage. The main nodes (bootstrap values > 0.6) are marked with pink triangles. RT = reverse transcriptase.

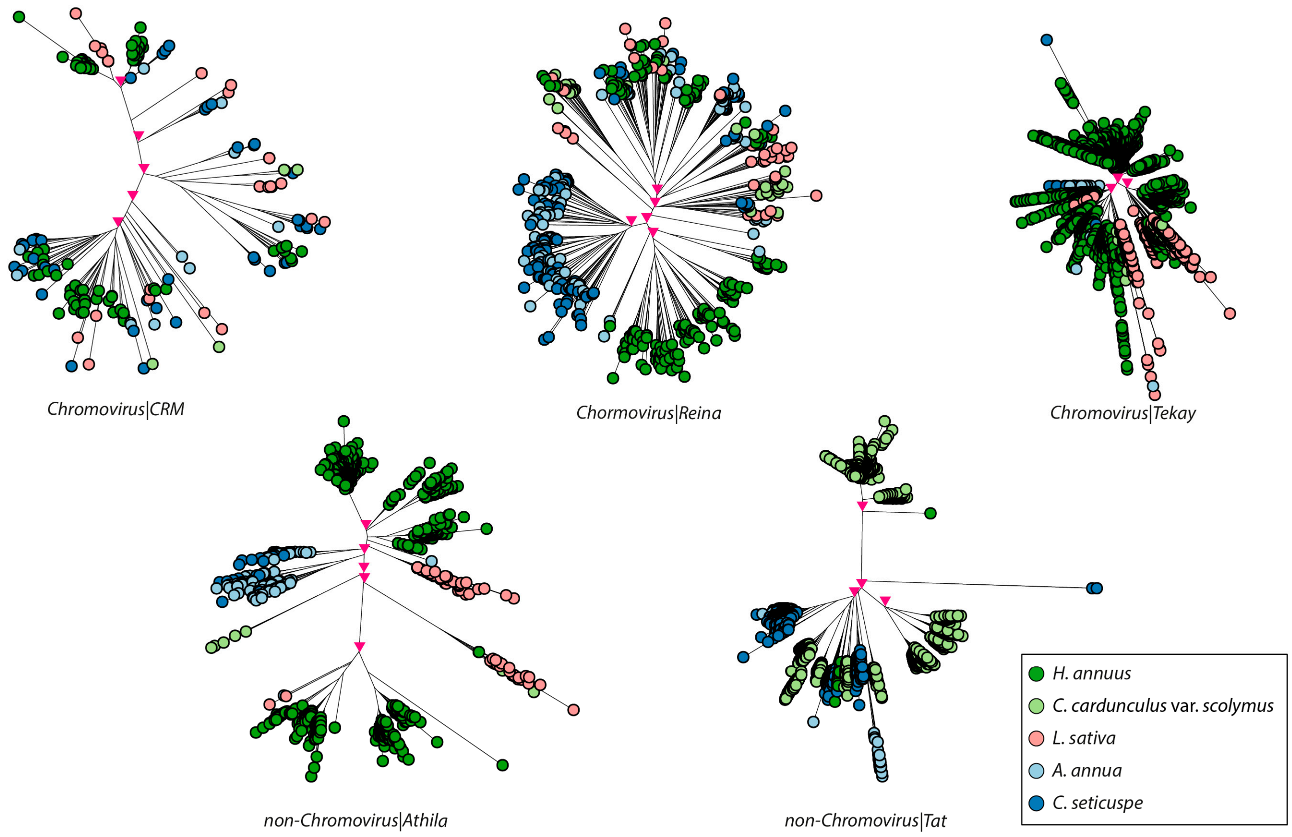

Figure S2. Phylogenetic trees of all RT sequences of *Gypsy* elements retrieved in the five Asteraceae species, separated according to the lineage. The main nodes (bootstrap values > 0.6) are marked with pink triangles. RT = reverse transcriptase.
